# Plan de egreso: herramienta del cuidado - diada con enfermedad crónica*

**DOI:** 10.15649/cuidarte.2754

**Published:** 2023-05-28

**Authors:** María Stella Campos-de-Aldana, Erika Yurley Durán-Niño, Silvia Liliana Ruiz-Roa, Astrid Nathalia Páez-Esteban

**Affiliations:** 1 . Universidad de Santander. Facultad de Ciencias Médicas y de la Salud. Instituto de Investigación Masira. Bucaramanga, Colombia. Email: al.campos@mail.udes.edu.co Universidad de Santander Universidad de Santander Facultad de Ciencias Médicas y de la Salud Instituto de Investigación Masira Bucaramanga Colombia al.campos@mail.udes.edu.co; 2 . Universidad de Santander. Facultad de Ciencias Médicas y de la Salud. Instituto de Investigación Masira. Bucaramanga, Colombia. Email: eri.duran@mail.udes.edu.co Universidad de Santander Universidad de Santander Facultad de Ciencias Médicas y de la Salud Instituto de Investigación Masira Bucaramanga Colombia eri.duran@mail.udes.edu.co; 3 . Universidad Francisco de Paula Santander, Cúcuta, Colombia. Email: silvialilianarr@ufps.edu.co Universidad Francisco de Paula Santander Universidad Francisco de Paula Santander Cúcuta Colombia silvialilianarr@ufps.edu.co; 4 . Universidad de Santander. Facultad de Ciencias Médicas y de la Salud. Instituto de Investigación Masira. Bucaramanga, Colombia. Email: ast.paez@mail.udes.edu.co Universidad de Santander Universidad de Santander Facultad de Ciencias Médicas y de la Salud Instituto de Investigación Masira Bucaramanga Colombia ast.paez@mail.udes.edu.co

**Keywords:** Alta Hospitalaria, Cuidado, Enfermedad Crónica, Patient Discharge, Care, Chronic Disease, Alta do Paciente, Cuidado, Doenga Crónica

## Abstract

**Introducción:**

La OMS reporta incremento de las enfermedades crónicas no trasmisibles, dada su complejidad, los pacientes presentan constantes hospitalizaciones, por lo cual es necesario fortalecer la competencia del cuidado de la enfermedad tanto del paciente como del cuidador en el hogar a través del programa plan de egreso.

**Objetivo:**

determinar el cambio en la competencia para el cuidado posterior a la implementación de herramienta plan de egreso para el cuidado en el hogar de la diada persona-cuidador familiar en situación de cronicidad en una institución de segundo nivel de atención.

**Materiales y métodos:**

Estudio de abordaje cuantitativo, seudo-experimental, con diseño de pruebas pre-test y pos-test, aplicando los instrumentos de “caracterización diada paciente-cuidador” y “Cuidar”. La población correspondió a 62 diadas. Muestreo no probabilístico a conveniencia. Se calculó del delta pre-post y se utilizó t-Student pareada para comparar.

**Resultados:**

el programa plan de egreso aumentó en el paciente las dimensiones de Conocimiento con un delta de 3.2 y Unicidad de 3,0 y competencia de cuidado global en 10,3; mientras que las competencias del cuidador familiar mejoraron en las dimensiones de Conocimiento con un delta de 3.34, Unicidad con delta 1.5, la dimensión de Disfrute con 1.7 y la de Reciprocidad -Relación con un delta de 1.61 para un total en la competencia de 9.2.

**Discusión y conclusiones:**

Estos hallazgos sugieren fortalecer el programa plan de egreso, durante la hospitalización y alta con el propósito de mejorar la competencia para cuidar en el hogar tanto del paciente como su cuidador familiar en situación de cronicidad.

## Abstract (en)

**Introduction:**

The WHO reports an increase in chronic non-communicable diseases. Given the complexity of these diseases, patients are constantly hospitalized; therefore, it is necessary to strengthen the competence of both patients and caregivers at home to take care of the disease using the program “discharge plan.”

**Objective:**

To determine the change in care competency following the implementation of the tool “discharge plan” in a second-level care facility for in-home care of the chronically ill person and family caregiver dyad.

**Materials and Methods:**

Quantitative, quasi-experimental study, with pre-test and post-test design, using the “Characterization of the patient-caregiver dyad” form and the “CUIDAR” instrument. The population consisted of 62 dyads. Non-probabilistic convenience sampling was used. Pre- and post-delta was calculated, and paired Student t-test was used for comparison.

**Results:**

The “discharge plan” program increased the patients' dimensions of Knowledge by a delta of 3.2, Uniqueness by 3.0, and total care competency by 10.3. Family caregivers'competencies improved in the dimensions of Knowledge by a delta of 3.34, Uniqueness by 1.5, Enjoyment by 1.7, and Reciprocity relationship by 1.61, for a total competency score of 9.2.

**Discussion and conclusions:**

These findings suggest that the discharge plan program must be strengthened during hospitalization and discharge to improve the homecare competency of chronically ill patients and their family caregivers.

## Resumo

**Introdujo:**

A OMS relata um aumento nas doengas crónicas nao transmissíveis, dada sua complexidade, os pacientes apresentam hospitalizagóes constantes, razao pela qual é necessário reforjar a competencia de atendimento tanto do paciente quanto do cuidador em casa através do programa do plano de alta.

**Objetivo:**

determinar a mudanga na competencia de prestagao de cuidados após a implementagao de uma ferramenta de plano de alta para o cuidado domiciliar da díade pessoa- família cuidadora em uma instituigao de cuidados de segundo nível.

**Materiais e Métodos:**

Um estudo de abordagem quantitativa, pseudo-experimental, com um projeto pré-teste e pós-teste, aplicando os instrumentos "caracterizagao da díade paciente-cuidador" e "Cuidar". A populagao correspondia a 62 díades. Amostragem de conveniencia nao-probabilística. O delta pré-post foi calculado e foi utilizado o par t-Student para comparagao.

**Resultados:**

o programa do plano de alta hospitalar aumentou as dimensóes do Conhecimento do paciente com um delta de 3,2 e a exclusividade de 3,0 e a competencia global de prestagao de cuidados em 10,3; enquanto as competencias dos cuidadores familiares melhoraram nas dimensóes do Conhecimento com um delta de 3,34, a exclusividade com delta 1,5, a dimensao de prazer com 1,7 e a relagao de reciprocidade com um delta de 1,61 para uma competencia total de 9,2.

**Discussao e conclusóes:**

Estas descobertas sugerem o fortalecimento do programa do plano de alta, durante a hospitalizagao e alta com o objetivo de melhorar a competencia para cuidar em casa tanto do paciente quanto de seu cuidador familiar em uma situagao de cronicidade.

## Introducción

Según la Organización Mundial de la Salud (OMS), las enfermedades crónicas no trasmisibles (ECNT) son patologías de larga duración y de progresión lenta, en la mayoría de los casos generan dependencia total o parcial, causando el 63% de las muertes en el mundo^1^. En América Latina las personas con enfermedad crónica son atendidas generalmente por un cuidador familiar, quienes frecuentemente no cuentan con la habilidad ni la competencia para asumir las responsabilidades de cuidado en el hogar y son ellos quienes satisfacen las necesidades básicas de la vida diaria, actúan como supervisores, proporcionan apoyo activo, toman decisiones y verifican el desarrollo de acciones. El número creciente de adultos mayores de 60 años y que padecen varias enfermedades crónicas aumenta la demanda de atención y apoyo a sus cuidadores familiares^2^.

En Colombia, las ECNT se han identificado como un problema de salud pública en los últimos 20 años, impactando en especial población con vulnerabilidad social y económica, por lo cual se ha constituido como prioritario a nivel mundial, e incluido dentro de los retos del milenio^3^.

La OMS y la Organización Panamericana de la Salud (OPS) han establecido políticas y objetivos programáticos que buscan prevenir y controlar las condiciones crónicas en las Américas. En este orden de ideas, una de las estrategias contemplada por el gobierno es ofrecer al paciente y cuidador familiar un programa educativo que incluya adherencia al tratamiento, conocimiento y manejo de la patología, continuidad de seguimiento o control médico y del equipo de salud, y cuidados ante situaciones de riesgo. Temática que se encuentra contemplada en el programa de transición y egreso hospitalario; este programa es una expresión de cuidado que adquiere relevancia en este contexto y en particular cuando en los sistemas de salud, el incremento del costo institucional indica que es necesario el paso de servicios de mayor a menor complejidad y de la institución de salud a la casa, pero se considera que aún no se cuenta con la preparación para asumir esta responsabilidad^4^.

Es de recalcar que este programa es útil e importante para que el paciente y su familiar, esté informado, cuente con soporte para su cuidado, logre tener mejor competencia para el cuidado y por ende esté más seguro dentro de su hogar; además, busca minimizar riesgos, evitar complicaciones innecesarias y asegurar la continuidad del tratamiento^4^^,^^5^.

En este sentido, la competencia para el cuidado en la casa u hogar se define teóricamente como la capacidad, habilidad y preparación que tiene la persona con enfermedad crónica y/o cuidador familiar para ejercer la labor de cuidado en el hogar, la cual comprende seis categorías que se describen bajo el acróstico CUIDAR: conocimiento, unicidad, instrumental, disfrutar (bienestar), anticipación y relación social^4^.

Sumado a esto el programa plan de egreso permite fortalecer la capacidad de cuidado del cuidador familiar, darle soporte para asumir su responsabilidad, mejorar su calidad de vida y capacidad de autocuidado, evitando cargas innecesarias que esta situación le pueda generar. Así mismo logra que las actuaciones de los profesionales de salud sean mejor informada, más preparada, coherente, que responda a las necesidades y expectativas del usuario y le garantice mayor seguridad en el cuidado y a su vez propende por disminuir la carga profesional por complicaciones evitables y reingresos o fallecimientos prevenibles.

Desde el punto de vista profesional, esta es una guía estandarizada que debe tener establecidos pasos o secuencias organizadas, previamente determinados para cuidar de manera segura e integral al paciente en la institución y al salir tenga continuidad terapéutica^6^. En instituciones de referencia en la atención de personas con enfermedades crónicas, el plan de transición-egreso para los pacientes es necesario, pero pueden en algunos casos recibir excesivos lineamientos para su cuidado y se enfrentaban a terapéuticas en extremo complejas.

El paciente necesita una instrucción y un acompañamiento para poder cuidar mejor su salud, y el cuidador familiar quien responde por el cuidado del paciente o debe ayudarle, requiere de manera adecuada un acompañamiento e instrucción que fortalezcan su competencia como cuidador e incluso su propia capacidad de cuidarse^7^. Por lo cual es necesario capacitar a los profesionales de salud encargados de dar estas orientaciones para fortalecer sus competencias al respecto, que tengan las herramientas para realizarlo, considerando que es una responsabilidad social, para todos los profesionales en salud al momento del egreso, pero en especial para el personal de enfermería, que usualmente es el último contacto al egreso.

Con relación al plan de egreso, un grupo de investigadores diseño y validó esta herramienta^8^obteniendo puntajes entre 0,67 y 0,94 para los criterios de claridad, precisión, comprensión, relevancia y pertinencia. Por otro lado, Melo y colaboradores aplicaron este plan y encontraron aumentos de 12,3 y 19,6 en los puntajes de la competencia del cuidado global tanto en el paciente con ECNT como cuidadores familiares, diferencias estadísticamente significativas^9^.

Es por ello que se connota la importancia de la implementación de un plan con adecuados procesos de acompañamiento y seguimiento de los pacientes con enfermedad crónica enmarcados en la competencia para el cuidado, que incluya aspectos para mejorar la calidad de vida, la seguridad en el cuidado, minimizar las complicaciones y los riesgos, mayor continuidad y adherencia a las terapéuticas ^10^^,^^11^_._

El objetivo del estudio fue determinar el cambio en la competencia para el cuidado posterior a la implementación de herramienta plan de egreso para el cuidado en el hogar de la diada persona- cuidador familiar en situación de cronicidad en una institución de segundo nivel de atención.

## Materiales y Métodos

Estudio de abordaje cuantitativo, seudo-experimental, con un diseño de pruebas pre-test y postest, con un grupo de prueba, en el que se determinó el cambio en la competencia para el cuidado por el programa plan de egreso en la competencia para el cuidado en el hogar de un grupo de personas con enfermedad crónica hospitalizadas y sus cuidadores familiares en la ESE San Juan de Dios, institución de segundo nivel de atención en salud ubicada en el municipio de Floridablanca, Colombia. La muestra fue a conveniencia de 62 diadas paciente-cuidador familiar de pacientes con enfermedad crónica hospitalizados en los servicios de Urgencias y Medicina Interna.

El muestreo fue no probabilístico, por conveniencia de la diada paciente-cuidador familiar. Entre los criterios de inclusión de pacientes, se encuentran que sea paciente con diagnóstico de ECNT igual o mayor a 6 meses, hospitalizado en la ESE San Juan de Dios de Floridablanca y con orden de egreso hospitalario. Asimismo, los criterios de inclusión de cuidadores familiares fueron: cuidadores mayores de 18 años y cuidadores familiares en el rol de cuidador en un tiempo igual o mayor a 3 meses.

El proceso de reclutamiento de la diada paciente-cuidador familiar, se dio en conjunto por el investigador principal y co-investigadores, docentes de la Universidad de Santander (UDES), una co-investigadora enfermera de la institución en alianza y los auxiliares de investigación quienes fueron estudiantes del programa de enfermería de la UDES. Inicialmente, se aplicó la prueba pre test, seguidamente se realizó la intervención, es decir, se ingresaron las personas y sus cuidadores familiares al programa “Plan de Egreso” y antes del alta del hospital se evaluó la competencia con la prueba pos-test tanto en pacientes como cuidadores familiares.

Se siguieron los siguientes pasos: Rutina de visita programada por día a los servicios de urgencias y hospitalización, convocatoria a las personas con ECNT y los cuidadores familiares para la presentación del proyecto, verificación de criterios de inclusión y exclusión de los participantes y aplicación del consentimiento informado escrito. La recolección de la información se llevó a cabo entre marzo de 2016 a mayo de 2017.

La valoración inicial del paciente y su cuidador se realizó con la aplicación de los instrumentos de recolección de la información: -Ficha de caracterización de la diada paciente - cuidador familiar (GCPC-UN-D©)^12^ que permitió obtener los datos de identificación y el nivel de dependencia, estado mental, nivel de sobrecarga y (TICS) Tecnología de la información y Comunicación y el instrumento -Competencia para el cuidado en el hogar versión paciente y cuidador familiar, versión larga^13^, éste evalúa el nivel de competencia de cuidado.

### Intervención educativa

Se realizó la intervención conforme al plan de egreso para la diada paciente-cuidador familiar previamente validado y descrito en la publicación de Carrillo y colaboradores en 2015^14^. De forma general, este plan comprende las siguientes etapas:

1. *Realizar valoración integral al paciente y al cuidador teniendo en cuenta el nivel de autonomía funcional y cognitiva.*
2. *Clasificar al usuario según grado de riesgo para el egreso con base en la complejidad del tratamiento y el nivel de vulnerabilidad del usuario al salir del hospital.*
3. *Brindar educación e instrucción al paciente y al cuidador. Se educó sobre manejo de la patología, adherencia al tratamiento, signos de alarma y factores de riesgo, asistencia a los controles por el personal de salud de forma individualizada, verificando que las instrucciones quedaran comprendidas y dedicando el tiempo necesario según cada caso.*
4. *Remitir a grupos de apoyo en la comunidad*
5. *Realizar seguimiento hasta por un mes después del egreso, seguimiento responsable con el apoyo de herramientas informáticas.*

Asimismo, se usó como apoyo para la intervención el folleto “Programa Plan de Egreso” y la lista de chequeo para el egreso según protocolo establecido por la institución, material visual y escrito debidamente validado para la población del estudio^15^. Además, a los pacientes que solicitaron se les entregó el folleto prevención de accidentes y primeros auxilios.

Una vez el paciente y cuidador familiar fueron intervenidos, se aplicó el instrumento “Competencia para el cuidado en el hogar versión paciente y cuidador familiar - versión larga, (pos-test) con el fin de obtener datos de la eficacia del programa plan de egreso.

### Instrumentos

Ficha sociodemográfica de caracterización de la diada paciente-cuidador familiar el GCPC-UN-D©. Fue propuesto por el Grupo de Cuidado al Paciente crónico y su familia de la Facultad de Enfermería de la Universidad Nacional de Colombia. Incluye información de características sociodemográficas, aspectos del cuidado (tiempo y dedicación), parentesco con la persona cuidada, redes de apoyo, soporte social, uso de tecnologías de información (TICs), estado mental del paciente y cuidador con SPMSQ, funcionalidad con PULSES, aspectos de espiritualidad y diagnósticos médicos del cuidador y del pacientes^12^.

El instrumento de competencia para el cuidado en el hogar desarrollado por el Grupo de Cuidado al Paciente Crónico y su Familia de la Facultad de Enfermería de la Universidad Nacional de Colombia que fue elaborado en base en las taxonomías NOC (Nursing Outcomes Classification) con aspectos relacionados para el cuidado por parte del paciente con ECNT y el cuidador familiar. Consta de 60 ítems, con respuestas en escala likert con puntuaciones que van de 1 a 4; donde 1 es nunca/casi nunca y 4 casi siempre/siempre. Contiene 6 sub-escalas así: conocimiento (1 al 10), unicidad (13 a 22), instrumental (23 al 30), disfrutar (31 a 42), anticipación (43 a 48), y relación social e interacción (49 a 60).

Los resultados de la competencia para el cuidado en el hogar se valoran con los siguientes puntajes posibles entre 20 y 60, rangos iguales o superiores a 40 indican una alta competencia. Puntajes iguales o inferiores a 39 suponen una baja competencia para el cuidado^13^.

### Procesamiento y análisis de la información

Se realizó doble digitación, validación y depuración de la información. Análisis descriptivo de las características de la población estudio y de la competencia para el cuidado. Variables cualitativa se presentan frecuencias absolutas y relativas, variables continuas se evaluó distribución normal con la prueba Shapiro Wilk y se reportó la media e intervalo de confianza (95%). Fueron usadas pruebas T-Student para muestras relacionadas pareadas para identificar diferencias entre la valoración pre y pos intervención educativa y evidenciada por el valor delta, magnitud de la diferencia entre éstas. Se consideraron diferencias estadísticamente significativas con un valor p<0.05. Para el análisis se utilizó el software estadístico Stata versión14.

### Aspectos éticos

La presente investigación cumplió con las pautas éticas para las investigaciones biomédicas establecidas por el Consejo de Organizaciones Internacionales de Ciencias Médicas (CIOMS), las Guías de las Buenas Prácticas Clínicas y la Resolución 8430 de1993 del Ministerio de Salud de la República de Colombia. Se respetaron los derechos de autor y propiedad intelectual de los instrumentos y estrategias de intervenciones empleadas, otorgando los respectivos reconocimientos y créditos a éstos. La información proporcionada por los pacientes fue tratada conforme a la legislación de protección de datos personales vigente.

Este proyecto fue aprobado por el comité de ética de la Universidad de Santander y de la institución en alianza. En este proceso fue revisado también por grupo de Investigadores de la facultad (Instituto MASIRA de la Universidad de Santander (UDES).

## Resultados

En cuanto a las características sociodemográficas, se evidencio que el mayor porcentaje de los pacientes pertenecen al género masculino con un valor de 52%, el 56% tiene edad mayor o igual a 75 años; así mismo, en nivel de escolaridad primaria, correspondiendo 68%, el 84% procede de la zona urbana, 58% de estrato socioeconómico dos. En cuanto al estado civil, un 53% es viudo, con una ocupación de permanencia en el hogar de 48% y otra ocupación el 39%, al referirse a la religión el 85% profesa la católica, con un compromiso religioso medio del 42% (Tabla 1).

El instrumento también caracteriza la muestra de 62 cuidadores familiares, donde se evidencia que el mayor porcentaje, pertenecen al género femenino en un 74%, 61% con edad entre los 36 a 59 años, es importante establecer que este ciclo de vida corresponde a una etapa productiva; respecto a la escolaridad el 45% tienen estudios de primaria, el 87% es de procedencia urbana y pertenecen al estrato socioeconómico dos el 48%. Por otra parte, el 40% es casado con porcentajes muy similares del 26 y 24% solteros y unión libre. Teniendo en cuenta que los cuidadores familiares el 50% la ocupación es el hogar, se le facilita ejercer éste rol. De forma similar que los pacientes, los cuidadores profesan la religión católica en un 79%; así mismo muestra que los más altos porcentajes en un 42% y 47% tienen un mediano y alto compromiso religioso (Tabla 1)

**Tabla 1:** Características sociodemográficas de los pacientes con enfermedad crónica y cuidadores familiares, ESE San Juan de Dios de Floridablanca, Colombia, 2017

Variable	Paciente n=62 %(n)	Cuidador n=62 %(n)
Género		
Masculino	51,61(32)	25,81(16)
Femenino	48,39(30)	74,19(46)
Edad		
18-35	3,23(2)	20,97(13)
36-59	9,68(6)	61,29(38)
60-74	30,65(19)	17,74(11)
>75	56,45(35)	0,00(0)
Escolaridad		
Analfabeta	25,81(16)	1,61(1)
Primaria	67,74(42)	45,16(28)
Secundaria	1,61(1)	33,87(21)
Técnico	0,00(0)	12,90(8)
Universitario	1,61(1)	6,45(4)
NR	3,23(2)	0,00(0)
Zona		
Urbana	16,13(10)	12,90(8)
Rural	83,87(52)	87,10(54)
Variable	Paciente n=62 %(n)	Cuidador n=62 %(n)
Estrato Socioeconómico		
1	27,42(17)	25,81(16)
2	58,06(36)	48,39(30)
3	14,52(9)	24,19(15)
4	0,00(0)	1,61(1)
Estado civil		
Soltero	11,29(7)	25,81(16)
Casado	22,58(14)	40,32(25)
Separado	3,23(2)	6,45(4)
Viudo	53,23(33)	3,23(2)
Unión libre	9,68(6)	24,19(15)
Ocupación		
Hogar	48,39(30)	50,00(31)
Empleado	1,61(1)	14,52(9)
Independiente	6,45(4)	20,97(13)
Estudiante	4,84(3)	4,84(3)
Otro	38,71(24)	9,68(6)
Religión		
Católica	85,48(53)	79,03(49)
Evangélica	8,06(5)	12,90(8)
Otra	4,84(3)	4,84(3)
NR	1,61(1)	3,23(2)
Compromiso religioso		
Alto	38,71(24)	41,94(26)
Medio	41,94(26)	46,77(29)
Bajo	19,35(12)	11,29(7)

Para evaluar la eficacia del programa plan de egreso, se calcularon las medias de los puntajes totales y por dimensiones de la competencia del cuidado pre y pos intervención, posteriormente se calcularon los deltas, como la diferencia entre la media pos y pre intervención; también, se realizaron pruebas T de Student para muestras relacionadas o pareadas dados que las medidas presentaron distribución normal. Se observó aumento de la competencia total para el cuidado en los pacientes (delta 10,3 valor p 0,01), a expensas de las dimensiones conocimiento (delta 3.1, p <0,01) y unicidad (delta 3,0, p <0,01), Tabla 2.

**Tabla 2:** Comparación de los niveles de competencia para el cuidado por dimensiones pre y pos intervención en pacientes con enfermedad crónica y sus cuidadores, ESE San Juan de Dios de Floridablanca, Colombia, 2017

Dimensión	Pre Media (IC95%)	Pos Media (IC95%)	Delta Media	Valor p
En Pacientes				
Conocimiento	14,00(12,08 a 15,92)	17,02(14,85 a 19,19)	3,02	<0,01
Unicidad	19,81(17,36; 22,26)	22,81(19,96; 25,66)	3,00	<0,01
Instrumental	11,65(10,17; 13,13)	12,83(11,04; 14,63)	1,18	0,1
Disfrutar	19,06(16,78; 21,35)	20,04(17,64; 22,44)	0,98	0,2
Anticipación	9,00(7,77; 10,23)	9,83(8,43; 11,23)	0,83	0,1
Relación e interacción	23,77(21,28; 26,27)	25,85(23,59; 28,12)	2,08	0,1
Competencia para el cuidado	97,29(86,55; 108,04)	108,40(96,93; 119,86)	11,11	<0,01
En Cuidadores				
Conocimiento	23,62(22,05; 25,19)	26,98(26,06; 27,90)	3,36	<0,01
Unicidad	28,82(27,41; 30,23)	30,30(28,99; 31,61)	1,48	<0,01
Instrumental	19,16(18,11; 20,21)	20,12(19,14; 21,10)	0,96	<0,01
Disfrutar	28,16(26,76; 29,56)	29,76(28,44; 31,08)	1,60	<0,01
Anticipación	14,63(23,57; 15,07)	14,90(14,11; 15,69)	0,58	0,04
Relación e interacción	30,38(28,92; 31,84)	31,62(30,22; 33,02)	1,24	<0,01
Competencia para el cuidado	144,46(138,52;15,04)	153,68(148,81;158,55)	9,22	<0,01

*valor p de la prueba t de Student para muestras pareadas

De forma similar, aumentó la competencia total del cuidador familiar (delta 9,2, p <0,01) y de las dimensiones de conocimiento (delta 3.3, p <0,01), unicidad (delta 1.5, p <0,01), instrumental (delta 1.1, p <0,01), disfrutar (1.73, p <0,01), anticipación (delta 0.5, p 0,01), relación e interacción (delta de 1.6, p <0,01), Tabla 2.

Lo anterior evidencia que existe conocimiento referente a la enfermedad, el tratamiento farmacológico, tratamiento no farmacológico y algunas medidas preventivas. La unicidad hace referencia a las características internas y herramientas que tiene tanto el paciente como el cuidador familiar para ejercer el respectivo rol, como afrontar el proceso de enfermedad y hacer más práctico las medidas de cuidado. En lo referente al cuidador en la dimensión de disfrutar, el cuidador refiere bienestar para entender y cómo afrontar la responsabilidad del cuidado en la dimensión anticipación y finalmente en la dimensión de relación e interacción el cuidador es un buen mediador entre la persona que cuida y el equipo de salud, puede detectar las necesidades de las personas que cuida y el paciente no expresa verbalmente y maneja adecuadamente la comunicación, teniendo un gran vínculo afectivo con su familiar a quien cuida.

En cuanto a la competencia para el cuidado en pacientes con ECNT la evaluación pre y pos intervención educativa se evidencio que el 81.5% que representaba la población con un nivel bajo disminuyo a 81% subiendo 4 puntos. El alto nivel inicial de los pacientes fue 1.6% pre intervención aumento a un 6.4% pos intervención.

Respecto al nivel de competencia en los cuidadores de pacientes familiares a la pre intervención se evidenció que en los tres niveles los porcentajes en similares con un 37.1%, 27.4% y 27.4% en nivel bajo, medio y alto respectivamente, datos que mejoraron considerablemente post-intervención y a la evaluación se encontró que hubo un el nivel de conocimiento mejoro en los tres niveles. En el nivel bajo el grupo de cuidadores mejora la competencia de un 37.1% a un 17.7%. Así mismo en las competencias media y alta aumentando su competencia de 27.4% a 33.9%, respectivamente.

## Discusión

Al comparar las características sociodemográficas de la diada paciente cuidador familiar con ECNT, se evidencia similares resultados a los reportados en otro estudio que evaluó 2231 diadas en las cinco regiones geográficas de Colombia, en lo referente al perfil, coincide con otras publicaciones en las cuales los cuidadores familiares son en mayor porcentaje de género femenino, adultos, con ocupación hogar, sin ningún tipo de alteración mental; condiciones que favorecen al cuidador familiar para ejercer éste rol^16^^,^^13^^,^^17^.

Cada una de las dimensiones que se evaluaron en la competencia en el hogar sin la intervención tanto en los pacientes como cuidadores familiares para el cuidado en el hogar, en el componente de conocimiento, el instrumental y el de anticipación se evidencia un avance significativo, donde cerca de la mitad (50%) de los participantes tienen niveles altos^16^^,^^18^^,^^13^. Por lo cual lo cual es necesario fortalecer la competencia para cuidar en el hogar de personas con ECNT en sus diferentes dimensiones.

Es de resaltar que siendo seis las dimensiones de la competencia del cuidado, con el logro del avance del conocimiento, se modificó significativamente la competencia del paciente en forma general. En cuanto a los cuidadores familiares las dimensiones de conocimiento, unicidad, instrumental, disfrute, anticipación, y relación aumentaron de forma significativa, competencias fundamentales en el cuidado del enfermo con enfermedades crónicas. En un estudio publicado por Tang y colaboradores también identificaron mejoría en los puntajes de competencias para el cuidado en cuidadores de pacientes con deterioro cognitivo, la cual fue medida con la escala CSI (Care Skill Inventory) posterior a la intervención, y 3 meses luego, pero su intervención fue enfocada en psicoeducación^19^.

En un estudio llevado a cabo en Taiwan con participación de pacientes que egresaban de una institución de salud realizan observaciones respecto de que en el plan de egreso para el alta hospitalaria se estaba incluyendo aspectos de educación, instrucciones para la supervisión de ejercicios, nutrición, monitoreo de medicamentos, prevención de secuelas, cuidado general y manejo de problemas, donde comparan cuando se enfatiza en estas instrucciones con el cuidado usual, esto en pacientes con fractura de cadera, la competencia para el cuidado la midieron con Caregiver Competence Scale, realizaron seguimiento hasta un año donde identificaron mejores puntajes en el grupo que intervinieron, mostrándose de esta forma también la importancia del diseño de planes de egreso según las necesidades de cada paciente y de que fueron intervenciones realizadas por enfermería^20^.

El estudio de Carrillo y colaboradores publicado en 2015, donde se utiliza también el instrumento que se presenta en esta investigación para medir la competencia del cuidado, con información de 959 participantes, refieren la dimensión con mayor proporción de altos niveles fueron instrumental (72.9%), relación (67.4%), anticipación(62.3%), Conocimiento (60.7%), unicidad (56.9%) y disfrutar(53.8%) 14, los reportes para la presente investigación se presentan como medidas de tendencia central para facilitar la comparación pre-pos, sin embargo se realizaron estos análisis y se identificó en la medición pre intervención con niveles altos en cada una de las dimensiones son: instrumental (3.2%), relación (16.1%), anticipación(4.8%), Conocimiento (11.3%), unicidad (3.2%) y disfrutar(25.8%), lo cual indica que fueron más bajos que los reportados por Carrillo y colaboradores, pero estes fue un estudio población ambulatoria (en sus domicilios), mientras que la población de la presente investigación fueron pacientes con ECNT y sus cuidadores familiares que egresaban de una institución de segundo nivel.

Una de las principales limitaciones del estudio es la ausencia de grupo control o de comparación, así como el muestreo por conveniencia. Por lo tanto, se debe tener precaución en la interpretación y aplicabilidad de los resultados, pues no se pueden atribuir solo al efecto de la intervención, puede haber potenciales variables de confusión. Este potencial sesgo se intentó reducir a través del análisis de pruebas estadísticas para medidas relacionadas o pareada, del tipo antes y después de la intervención en la misma población. Además, los resultados deben ser extrapolados con precaución dado a la limitada validez externa, dado que sólo representan a la muestra descrita en este artículo (validez interna).

En este sentido, se recomienda avanzar en esta línea de investigación y realizar ensayos clínicos aleatorizados y controlados para evaluar la eficacia de la intervención en personas con ECNT y sus cuidadores familiares en un escenario más amplio, con características basales similares y ajustando por el puntaje de competencia inicial, además de realizar un seguimiento para identificar el efecto de la intervención en un periodo de tiempo más amplio.

Asimismo, se recomienda fortalecer el plan de egreso hospitalario a través de educación relevante y pertinente de enfermería con el propósito de mejorar la competencia del cuidado domiciliario, control de la enfermedad crónica y disminuir sus complicaciones.

## Conclusiones

La intervención educativa desarrollada en el plan de egreso de pacientes con ECNT mejoraron la competencia para el cuidado de manera significativa en todas las dimensiones en el cuidador. En los pacientes esta mejoría fue significativa para conocimiento y unicidad, aunque se aprecia que los puntajes competencia del cuidado tienden a mejorar, no fue totalmente significativo en las dimensiones de instrumental, disfrutar, anticipación y relaciones e interacciones. De esta forma se documenta los beneficios del programa plan de egreso como una herramienta eficaz para la diada en situación de cronicidad.
